# Long-read sequencing disentangles isoform complexity at allele-specific loci

**DOI:** 10.1038/s41598-025-97362-z

**Published:** 2025-11-11

**Authors:** Lison Lemoine, Sarah Hoelzl, Tim P. Hasenbein, Elisabeth Graf, Daniel Andergassen

**Affiliations:** 1 Institute of Pharmacology and Toxicology, Technical University of Munich, Munich, Germany; 2 DZHK (German Centre for Cardiovascular Research), Partner Site Munich Heart Alliance, Munich, Germany; 3 Institute of Human Genetics, Klinikum Rechts Der Isar, Technical University of Munich, Munich, Germany

**Keywords:** Data processing, Epigenomics

## Abstract

In recent years, long-read sequencing technologies have detected transcript isoforms with unprecedented accuracy and resolution. However, it remains unclear whether long-read sequencing can effectively disentangle the isoform landscape of complex allele-specific loci that arise from genetic or epigenetic differences between alleles. Here, we combine the PacBio Iso-Seq workflow with the established phasing approach WhatsHap to assign long reads to the corresponding allele in polymorphic F1 mouse hybrids. Upon comparing the long-read sequencing results with matched short reads, we observed general consistency in the allele-specific information and were able to confirm the imprinting status of known imprinted genes. We then explored the complex imprinted *Gnas* locus known for allele-specific non-coding and coding isoforms and were able to benchmark historical observations. This approach also allowed us to detect isoforms from both the active and inactive X chromosomes of genes that escape X chromosome inactivation. The described workflow offers a promising framework and demonstrates the power of long-read transcriptomic data to provide mechanistic insight into complex allele-specific loci.

## Introduction

Mammals are diploid and, therefore, have two copies of each chromosome, one inherited from each parent. Given that both gene copies on the parental alleles are regulated independently, genes can be expressed from both alleles, known as biallelic expression, or from one allele, known as allele-specific expression. Allele-specific expression can arise due to genetic or epigenetic differences between the alleles, with a substantial proportion attributed to genetic differences^1–3^. These allelic genetic variations have been demonstrated to induce allele-specific expression by influencing transcription factor binding or impacting post-transcriptional processes in an allele-specific manner^3–5^. Genomic imprinting is a prominent example of allele-specific expression arising from epigenetic differences between alleles^6^. Mechanistically imprinted genes are typically clustered, with allele-specific silencing regulated by differential DNA methylation^6,7^. For several imprinted clusters, the unmethylated imprinted control element acts as a promoter for a long non-coding RNA (lncRNA) that silences nearby genes by targeting histone-modifying complexes^7,8^. These genes play a crucial role in various biological processes, such as fetal development and homeostasis, and their dysregulation is associated with several diseases^9,10^. Another classic example of an epigenetic phenomenon leading to allele-specific expression is X chromosome inactivation (XCI), a process critical for dosage compensation between the sexes^11^. During XCI, one of the female X chromosomes undergoes epigenetic silencing facilitated by the lncRNA *Xist*, which is expressed exclusively from the future-inactive X chromosome^12,13^. Interestingly, some genes can escape this process and are consequently expressed from both X chromosomes^14–16^.

Current approaches for mapping allele-specific expression primarily rely on short-read sequencing data^1,17–20^. In mice, these approaches depend on single nucleotide polymorphisms (SNPs) between genetically distinct inbred strains to assign sequencing reads to the corresponding parental allele^2^. This strategy allowed for a nearly complete map of the imprinted landscape, spanning a comprehensive set of murine organs and developmental stages^5,18,21,22^. A limitation of short-read sequencing is its inability to accurately quantify entire allele-specific isoforms, a characteristic of imprinted loci, which often harbor multiple overlapping allele-specific isoforms. A prime example of such a complex locus is the imprinted *Gnas* cluster, where the same gene locus is transcribed into distinct biallelic, maternal, and paternal isoforms^23,24^. Dysregulation of the *Gnas* cluster can lead to growth defects and pseudohypoparathyroidism^23^. Hence, investigating the isoform landscape of disease-relevant loci displaying diverse isoform profiles can illuminate their underlying mechanisms and functions. Recent advances in long-read sequencing technologies, such as the Oxford Nanopore and Pacific Biosciences (PacBio) platforms, have allowed for transcriptomic analysis with a high resolution, span, and accuracy^25,26^. Long-read sequencing can span challenging genomic regions and capture full-length transcripts, enabling a comprehensive gene expression analysis^27,28^. However, allele-specific analysis of long-read data poses unique challenges due to the need for specialized bioinformatic approaches that differ from those used for short-read sequencing analysis, as recently shown in a large allele-specific human study^29^. Here, we present a user-friendly framework for analyzing allele-specific expression using long-read sequencing. To accomplish this, we analyzed the whole brain of a highly polymorphic F1 mouse by integrating the PacBio Iso-Seq pipeline with the established phasing approach WhatsHap^30,31^. This strategy allowed us to capture and quantify the allele-specific isoform diversity of protein-coding genes and lncRNAs. Upon comparing the long-read sequencing results with organ- and background-matched short-read sequencing data, we observed a general consistency in the allele-specific expression data across most genes. We further highlight the effectiveness of the described workflow by benchmarking historical observations at the imprinted *Gnas* locus and known XCI escape genes. In summary, the described workflow offers a promising framework for future studies aiming to shed light on loci with allele-specific isoform diversity.

## Results

### Assigning long reads to the corresponding allele

**Fig. 1:**
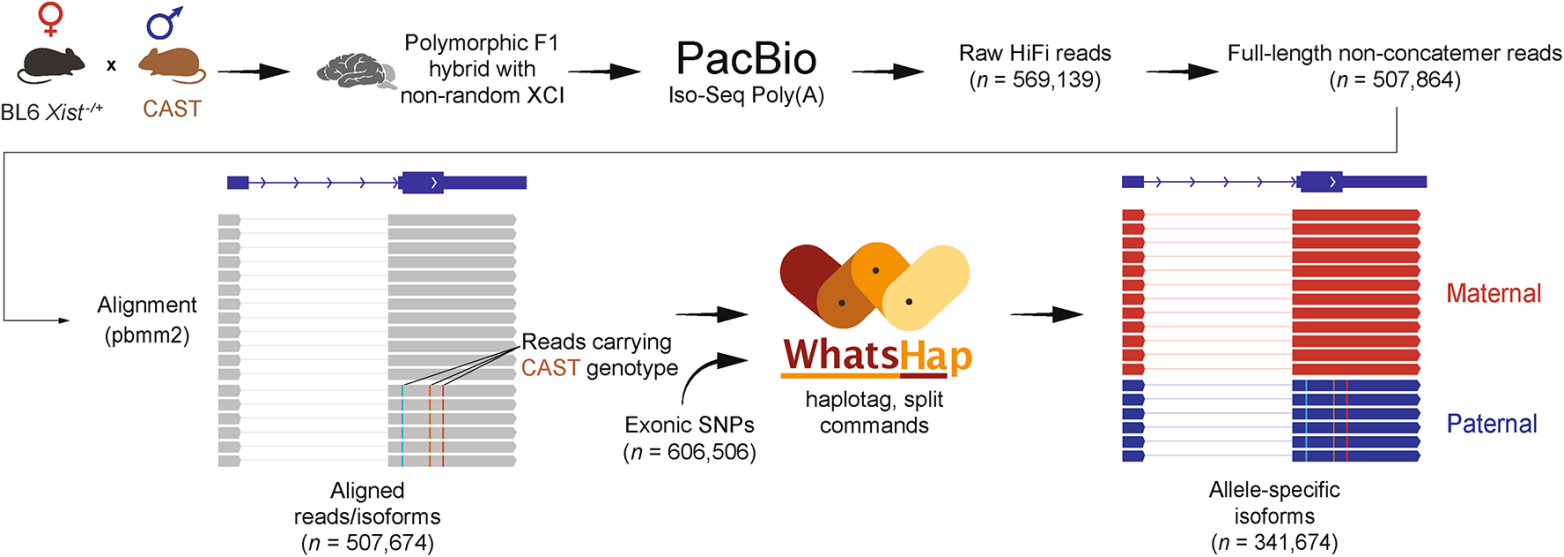
Integrating PacBio Iso-Seq with WhatsHap for allele assignment in a polymorphic F1 mouse hybrid. The raw reads obtained from long-read sequencing are filtered using the Iso-Seq pipeline. These filtered reads are called full-length non-concatemer reads and are aligned using PacBio’s minimap2 wrapper. Using known exonic SNPs between BL6 and CAST^2^, long reads are assigned to the corresponding allele (maternal or paternal).

To capture the allele-specific expression landscape through long-read sequencing, we bred C57BL/6J (BL6) females heterozygous for a deletion of the proximal A-repeat of *Xist* (*Xist*^−/+^) with CAST/EiJ (CAST) males (Fig. 1)^32^. Next, we collected a whole adult brain from a resultant F1 female hybrid (BL6^ΔXist^ × CAST) and applied the PacBio technology-based Iso-Seq workflow. This poly-A selection-based method allows for a comprehensive exploration of isoform discovery at the gene level in the transcriptome (see "Methods"). The resulting dataset enables the detection of allele-specific expression for autosomal genes and X-linked genes due to the *Xist* deletion, preventing XCI of the maternal BL6 allele and resulting in a fully skewed XCI pattern. A total of 569,139 HiFi reads were recovered, with an average read length of 2836 base pairs. These reads were further processed and filtered, resulting in 507,864 full-length non-concatemer reads, defined as reads that contain all essential transcript features, including both 5’ and 3’ primers, a poly-A tail, and the full insert sequence with no truncations or internal priming artifacts. The long reads were aligned to the reference genome using pbmm2, a PacBio wrapper of Minimap2, resulting in 507,674 uniquely aligned reads^33^.

**Fig. 2:**
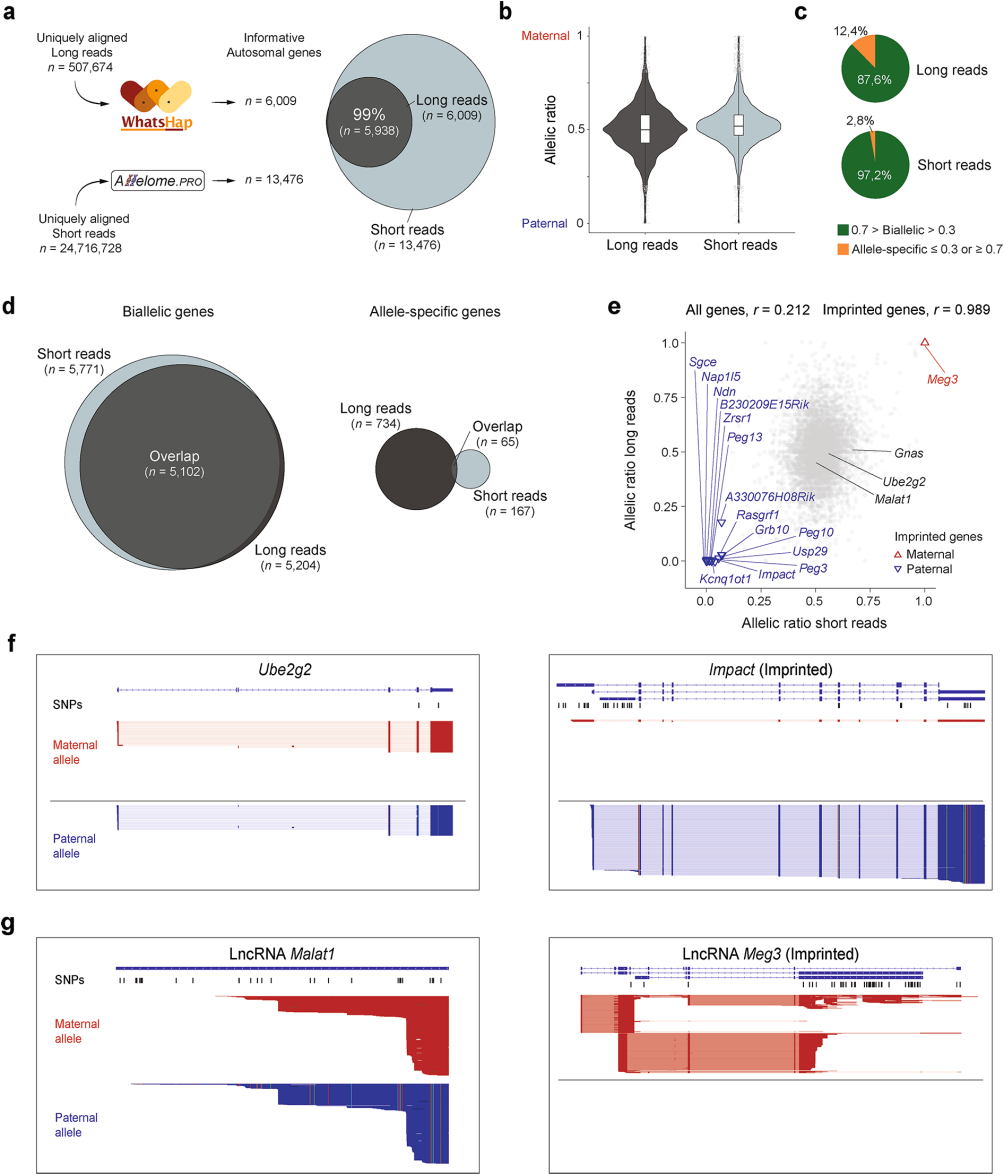
Comparative validation of allele-specific long-read results using short-read sequencing results. (**a**) Number of uniquely aligned reads used as input for allele-specific analysis and the number of informative genes in short (reads overlapping a SNP ≥ 20) and long (reads overlapping a SNP ≥ 10) reads. High overlap of informative autosomal genes between the datasets is shown with a Venn diagram. Long reads are represented in dark gray, and short reads are represented in light gray. (**b**) Violin plots of the allelic ratios of autosomal genes from the long- and short-read analysis. In our setup, allelic ratios range from 0 to 1 (0 = 100% expression from the paternal allele, 1 = 100% expression from the maternal allele). (**c**) Pie charts showing the proportion of biallelic genes (0.3 < allelic ratio < 0.7) and allele-specific genes for the overlapping autosomal genes between the long- and short-read datasets. (**d**) Venn diagrams showing the overlap of genes labeled as biallelic (left) and allelic (right) in both datasets. (**e**) Pearson correlation of short- and long-read allelic ratios for all genes (gray, opacity 20%) and for known imprinted genes (*n* = 15). Known imprinted genes are indicated in blue (paternally expressed) or red (maternally expressed), while genes labeled in black are presented in other figure panels. (**f**) Genome browser visualization showing the long-read data for the biallelic gene *Ube2g2* and the known paternally expressed imprinted gene *Impact*. Maternal reads are indicated in red, and paternal reads are indicated in blue. SNPs used to assign long reads to the BL6 or CAST allele are indicated in black. To visualize the long reads in the IGV browser, the following options were used: the ‘Link supplementary alignments’, represented by a thin blue/red connecting line, and ‘Hide small indels’. Black lines indicate regions of the primary alignment that span splice junctions. No reads have been collapsed, and all reads aligned to each locus are shown. (**g**) Genome browser visualization showing the long-read data for the biallelic lncRNA *Malat1* and the known maternally expressed imprinted lncRNA *Meg3*.

To investigate allele-specific expression using long-read sequencing, we applied the established phasing method, WhatsHap, using the haplotag and split subcommands^30,31^ (see "Methods" and Supplementary Methods). Using available exonic SNP information between BL6 and CAST (*n* = 606,506)^2^, the WhatsHap tool was used to assign long reads to maternal or paternal alleles. A single SNP was sufficient to map long reads to the corresponding alleles, as shown for the paternally expressed imprinted gene *Ndn* (Supplementary Fig. 1). Of the 341,674 tagged long reads, 52.4% (*n* = 179,197) were assigned to the maternal BL6 allele, and 47.5% (*n* = 162,477) were assigned to the paternal CAST allele (Fig. 1), covering 13,732 genes. A minimum cutoff of 10 reads overlapping at least one SNP per gene was applied, resulting in 6203 informative genes (coding genes = 6123, non-coding genes = 80), including 6009 autosomal and 194 X-linked genes (Fig. 2a, Supplementary Table S1, sheet a). In summary, we combined the PacBio Iso-Seq workflow with established phasing methods, allowing us to explore the allele-specific expression landscape in highly polymorphic F1 hybrids using long-read sequencing.

### The allelic status shows general consistency between short- and long-read sequencing, with the highest confirmation for imprinted genes

After implementing our long-read sequencing approach for allele-specific expression analysis, we focused on validating the allelic long-read landscape. To accomplish this, we conducted a comparative analysis with a background-matched short-read dataset, utilizing an adult F1 brain sample in the BL6xCAST genetic background^18^. We conducted an in-depth analysis using the established Allelome.PRO approach, a pipeline for defining allele-specific expression in short-read sequencing^1^, with 24,716,728 uniquely aligned short reads as input (Fig. 2a, see "Methods"). We recovered allelic information for 13,476 autosomal genes (reads overlapping a SNP ≥ 20) and found that of the 6009 autosomal genes detected by long-read sequencing, 99% (*n* = 5938) overlapped (Fig. 2a, Supplementary Table S1, sheet a-c). Among the genes that overlapped, most autosomal genes exhibited biallelic expression with both long and short reads (Fig. 2b). We used the previously defined allelic ratio cutoff of ≤ 0.3 or ≥ 0.7 to classify expression as paternal or maternal, respectively, while all other cases were classified as biallelic, a threshold shown to effectively distinguish known imprinted genes from biallelically expressed genes^1^. Within the 5204 and 5771 biallelic genes identified in the long-read and short-read datasets, respectively, 98% (*n* = 5102) overlapped. (Fig. 2c and d). However, there was a notable difference in the proportion of allele-specific genes. For short reads, 2.8% (*n* = 167) of the genes showed allele-specific expression, while a higher proportion of genes, 12.4% (*n* = 734), was detected in the long-read dataset, with 65 genes present in both datasets (Fig. 2c and d, Supplementary Table S1, sheet d). Of these shared 65 allele-specific genes, the majority had the same allelic direction (*n* = 58, 89.23%). Among these, 15 are known imprinted genes in the brain whose imprinted status was correctly determined^5^. The remaining seven genes showed different patterns due to the following observations. *Trappc9* suffers from the lack of strand specificity in short-read data due to its overlap with the paternally expressed imprinted gene *Peg13*. *Gm5424* is a highly repetitive retroposed gene, which may introduce sequencing and alignment bias. The remaining genes (*Sdccag8*, *Sox8*, *Ogdhl*, *Csnk2a1*, and *Churc1*) can be explained as genes expressed slightly below or above the allelic ratio cutoff. Overall, the correlation of the allelic ratio for all informative genes shared between short- and long-read sequencing was positive but relatively weak (*r* = 0.212, *p* = 2.2 × 10^−16^, Fig. 2e). However, we observed a markedly high correlation (*r* = 0.989, *p* = 2.989 × 10^−12^, *n* = 15) for known imprinted genes in the brain (Fig. 2e). To illustrate the power of this approach, we visualized the biallelically expressed gene *Ube2g2* (*n* = 29 maternal and 28 paternal long reads) and the paternally expressed imprinted gene *Impact* (*n* = 2 maternal and 217 paternal long reads, Fig. 2f). This approach also enabled us to correctly assign the allele-specific status of lncRNAs, including the biallelically expressed lncRNA *Malat1* (*n* = 598 maternal and 705 paternal long reads) and the maternally expressed imprinted lncRNA *Meg3* (*n* = 737 maternal and 0 paternal long reads, Fig. 2g). In conclusion, we observed general agreement in allelic status between short- and long-read sequencing data, with the highest level of confirmation for biallelic and imprinted genes.

### Allele-specific long-read analysis of the *Gnas* cluster benchmarks historical observations

**Fig. 3:**
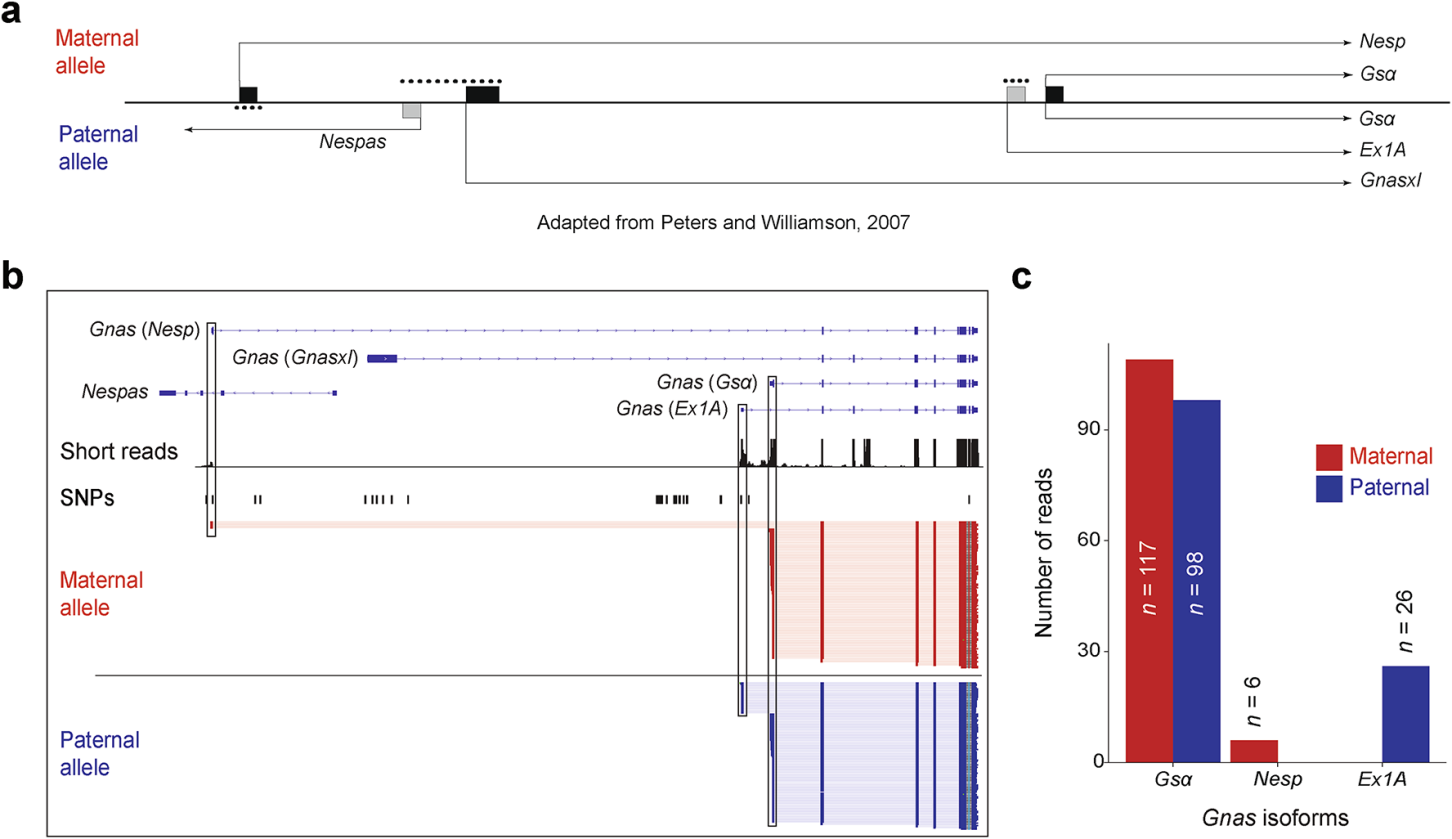
Allele-specific long-read analysis of the *Gnas* cluster (**a**) Organization of the *Gnas* cluster, adapted from Peters and Williamson^23^. While many transcripts have been identified within this cluster, only the most notable isoforms are shown for simplicity. Dots indicate methylated promoters on either the maternal or paternal allele. First exons of protein-coding (black rectangles) and non-coding isoforms (gray rectangles) are shown. (**b**) Genome browser tracks at the *Gnas* locus showing long-read data for the maternal (red) and paternal (blue) alleles as well as short-read data. SNPs used to assign long reads to the BL6 or CAST allele are indicated in black. (**c**) Quantification of *Gnas* isoform counts for the maternal and paternal alleles.

To further highlight the strength of the allele-specific long-read analysis, we conducted a detailed inspection of the imprinted *Gnas* cluster. At least 50 different transcripts derived from alternative promoters have been identified in the *Gnas* cluster with multiple allele-specific isoforms^23,24,34^. The most notable *Gnas* gene products include the biallelically expressed *Gsα* isoform, the paternally expressed isoforms *Gnasxl* and *Ex1A*, and the maternally expressed *Nesp* isoform. In addition, the *Gnas* locus encodes the paternally expressed antisense lncRNA *Nespas* (Fig. 3a)^23^.

In our long-read data, we detected and recovered allele-specific information from three isoforms of the *Gnas* cluster, namely, *Gsα*, *Nesp*, and *Ex1A* (Fig. 3b). The *Nespas* and *Gnasxl* isoforms are not expressed in the adult brain, supported by short- and long-read sequencing data (Fig. 3b). We identified *Gsα* as biallelically expressed with 117 maternal and 98 paternal reads, consistent with previous observations (Fig. 3c)^23^. The imprinted *Nesp* isoform was correctly identified as maternally expressed, with all six reads coming from the maternal allele. Similarly, we confirmed paternal expression of *Ex1A* by detecting all 26 reads exclusively from the paternal allele. In summary, our allele-specific long-read analysis of the *Gnas* cluster validates previous findings and underscores the effectiveness of this approach in providing mechanistic insights within imprinted loci characterized by overlapping allele-specific isoforms.

### Allele-specific long-read sequencing enables the detection of escape gene isoforms from both the active and inactive X chromosomes

**Fig. 4:**
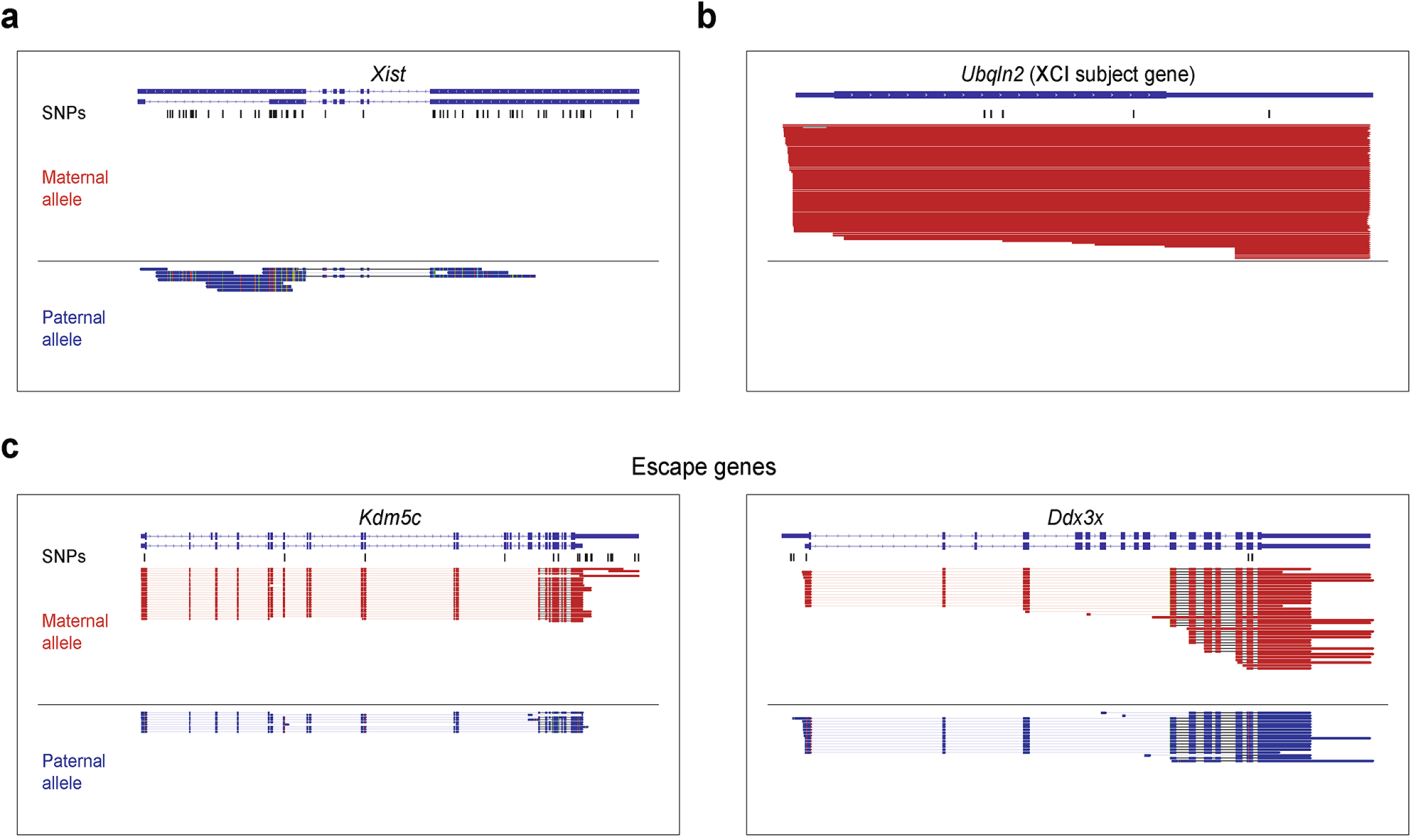
Long-read sequencing enables allele-specific assignment of known X chromosome inactivation escape genes. (**a**) Genome browser visualization of long-read data for *Xist*. (**b**) Genome browser visualization of long-read data for X-linked genes showing the non-escape gene *Ubqln2*. (**c**) Genome browser visualization of long-read data for the known escape genes *Kdm5c* and *Ddx3x*. SNPs used to assign long reads to the BL6 or CAST allele are indicated in black.

We then examined the allele-specific long-read landscape on the X chromosome by using our skewed XCI model caused by the *Xist* deletion (BL6^Δ*Xist*^ × CAST), which results in the expression of the maternal BL6 X chromosome (see "Methods")^32^. For all informative X-linked genes, we observed 12,623 long reads aligned to the X chromosome. The vast majority originated from the maternal active X chromosome, with only 69 (0.54%) aligning to the paternal inactive X chromosome. Of these, 39 reads correspond to *Xist* and the two known escape genes, *Ddx3x* and *Kdm5c*. As anticipated by our model, *Xist* was found to be exclusively expressed from the inactive X chromosome (*n* = 10 paternal long reads, Fig. 4a), resulting in maternal expression of the majority of X-linked genes (*n* = 191/194, allelic ratio > 0.9, Supplementary Table S1, sheet a). For instance, the *Ubqln2* gene is subject to XCI and displays exclusively maternal reads (*n* = 72 maternal and 0 paternal reads), which is in line with the findings in previous reports^30,35^ (Fig. 4b). Furthermore, we examined the isoforms of well-established genes known to escape XCI, such as *Ddx3x* and *Kdm5c*^36^ (Fig. 4c). As expected, we detected biallelic expression of these escape genes. Specifically, for *Ddx3x*, we identified 36 maternal reads and 18 paternal reads, while for *Kdm5c*, we observed 26 maternal reads and 11 paternal reads. This approach also sheds light on whether isoform differences can be detected depending on whether a gene is transcribed from the active or the epigenetically silenced X chromosome. For the two escape genes detected in our long-read data, no apparent isoform differences were observed between the active and inactive X chromosomes (Fig. 4c). In conclusion, the described workflow demonstrates the feasibility of detecting escape genes using long-read sequencing and enables the investigation of whether the inactive X chromosome influences isoform patterns.

## Discussion

In this study, we combined PacBio long-read sequencing with an established phasing approach, WhatsHap, to capture the allele-specific expression landscape of autosomal and X-linked genes^26,30,31^. To this end, we performed long-read sequencing on an adult brain isolated from a highly polymorphic F1 mouse, allowing us to use SNPs to assign long reads to the corresponding allele. To benchmark the allele-specific long-read results, we compared them to short-read sequencing analysis from a background-matched brain^18^. Overall, we observed a high degree of overlap for biallelic and imprinted genes, representing the largest proportion of informative genes. In contrast, we observed a notable difference in the proportion of allele-specific genes, likely driven by genetic differences between the alleles. Specifically, a higher proportion of allele-specific genes was found in the long-read dataset, which may be attributed to the ability of long-read sequencing to detect full-length isoforms. Several other factors could also contribute to these variations, including the lack of strand-specific information in the short-read comparison, leading to false positive allelic calls due to overlapping transcripts and differences in read coverage, and technical explanations such as alignment biases. Considering that disagreement was found only for allele-specific non-imprinted genes, which generally tend to have a lower allelic bias than imprinted genes, the variation may also be attributed to genes that fluctuate around the allelic ratio cutoff. Thus, for future studies, it is recommended to incorporate additional replicates to enhance the robustness and reproducibility of allele-specific expression detection, as previously suggested^37,38^.

Nonetheless, we were able to accurately assign the allele-specific status of known imprinted genes, including lncRNAs. We highlight the power of this approach by exploring allele-specific isoforms in the imprinted *Gnas* cluster. Our allele-specific long-read quantification confirmed the allelic status of the biallelically expressed *Gsα*, maternal *Nesp*, and paternal *Ex1A* isoforms, emphasizing the potential of this approach to elucidate the mechanism of complex allele-specific loci. To identify novel imprinted genes using long-read sequencing, a reverse cross would be required. This approach would help determine whether allele-specific expression arises from imprinting or caused by genetic differences between the alleles^1,5,18^. Furthermore, we correctly assigned the majority of X-linked genes as exclusively expressed from the maternal allele, as expected from our model. We also identified the lncRNA *Xist* as being expressed from the paternal inactive X chromosome, along with known escape genes showing the expected expression from both the active and inactive X chromosomes. Interestingly, no obvious isoform differences were detected for these escape genes, regardless of whether they were expressed from the active or inactive X chromosome, suggesting that the repressive epigenetic signature of the Barr body does not give rise to inactive X-specific isoforms.

Future studies should use long-read sequencing across multiple organs, cell types, and developmental timepoints to map the allele-specific landscape, as this approach provides critical insights into the regulation of gene expression at the isoform level. Using the described framework, these findings could be extended to humans when phased SNP information is already available. Otherwise, SNP calling and chromosome-wide phasing would be required for each human sample to accurately distinguish between alleles. For such challenging datasets, more complex pipelines like LORALS can enable detailed transcriptomic analyses, including classification of alternative transcript structure, rare variant effects, and isoform usage^29^. In summary, we provide a user-friendly framework to encourage future studies to adopt allele-specific long-read analysis, facilitating the disentangling of complex loci and offering mechanistic insights into gene regulation.

## Methods

### Mouse model

We obtained C57BL/6J (BL6) females carrying a heterozygous deletion of the proximal A-repeat (*Xist*^−/+^) from Riken BRC Japan (RBRC02655: B6;129-Xist < tm5Sado >) and crossed them with a wild-type CAST/EiJ (CAST) male (JAX: Strain #000928)^32^. From the resulting highly polymorphic F1 offspring (BL6^Δ*Xist*^ × CAST), we isolated the whole adult brain (9 weeks) of a female and promptly snap-froze it, storing it at − 80 °C for subsequent use.

### PacBio Iso-Seq library preparation and sequencing

RNA was extracted from the whole brain according to the manufacturer’s instructions for Invitrogen TRIzol reagent. RNA concentration and purity were measured using a NanoDrop™ One spectrophotometer (Thermo Fisher Scientific). The integrity of the total RNA was assessed on an Agilent 4200 TapeStation System (Agilent Technologies) using an RNA Screentape analysis kit. The RNA was of high quality, with a RIN greater than 9. cDNA libraries were prepared according to the Procedure & Checklist—Iso-Seq™ Express Template Preparation for Sequel® and Sequel II Systems protocol. Briefly, 300 ng of total RNA was used to generate full-length cDNA using oligo-dT primers and the NEBNext Single Cell/Low Input cDNA Synthesis & Amplification Module (New England Biolabs). cDNA amplification was performed, followed by end repair and adapter ligation. Primer and polymerase binding was performed according to the SMRT Link Sample Setup using the Sequel II Binding Kit 2.1. The entire pool was sequenced on one SMRT cell on the Sequel II sequencing platform using the Sequel II Sequencing Kit 2.0 (Pacific Biosciences) with a 4-h pre-extension and 20-h run time.

### Long-read data processing and allele-specific analysis

Raw long reads were demultiplexed and processed using the PacBio standard pipeline (SMRT Link version 11.0). The demultiplexed brain sample yielded 569,139 long reads with an average length of 2836 bp and read quality of Q40. These reads were further processed and filtered, resulting in 507,864 full-length non-concatemer reads, which were uniquely aligned to the mm10 genome using pbmm2 (version 1.10.0), a minimap2 (version 2.15) wrapper for PacBio^33^.

To conduct allele-specific long-read analysis, each read was individually tagged using the ‘haplotag’ command from WhatsHap (version 1.7.0)^30,31^. This tagging process involved incorporating exonic SNPs (*n* = 606,506, exons annotated from RefSeq) obtained from the Sanger database, specifically those differentiating between CAST and BL6^2^. The resulting BAM file was then separated into two haplotype BAM files, one for CAST and one for BL6, using the ‘split’ command from WhatsHap. Genes were visualized in the BAM files of the two haplotypes using the Integrative Genomics Viewer (version 2.16.0) with the following options: ‘hide indels,’ and ‘link supplementary alignments’^39,40^.

To compute the allelic ratio for every gene, the long-read counts were first quantified for both haplotype BAM files using htseq-count for every gene within the RefSeq annotation (downloaded February 2018) with the stranded option (HTSeq version 0.12.4)^41^. Subsequently, we computed allelic ratios for every informative gene with at least 10 reads overlapping at least one exonic SNP. For this computation, we divided maternal reads by the total (maternal and paternal reads) for each gene, resulting in a ratio of 1 for maternally expressed genes and 0 for paternally expressed genes. This analysis was performed using R (version 4.2.3).

### Short-read data processing and allele-specific analysis

Short-read data (generated from Illumina RNA-Seq TruSeq v3) was downloaded for a male F1 adult brain (BL6xCAST) (Sequence Read Archive SRP020526)^18^. The short reads were aligned to the mm10 genome using STAR, excluding alignments with an intron size over 100,000, non-canonical junctions, or that were multimapped (version 2.6.0c)^42^. We performed allele-specific expression analysis for informative genes, defined as having at least 20 reads overlapping a SNP, using the Allelome.PRO pipeline with the exonic SNP file as input^1,2^.

## Supplementary Information

Supplementary Material 1.

Supplementary Material 2.

## Data Availability

The long-read Hi-Fi raw reads (brain_flnc.bam) for the whole brain, together with the processed maternal and paternal long-reads BAM files generated for this study, have been deposited in the Gene Expression Omnibus (GEO) and are accessible under GSE246857. The whole-brain short-read sequencing dataset was downloaded from the Sequence Read Archive (SRA) under the following accession number: SRP020526. Access to the allele-specific long-read resource is provided via the Integrative Genomics Viewer at: https://github.com/AndergassenLab/Allelic_Longreads.
