# Supplementary material for: Long-read sequencing disentangles isoform complexity at allele-specific loci
Source: Sci Rep. 2025 Nov 11;15:39389. doi: 10.1038/s41598-025-97362-z (PMC12606347; doi:10.1038/s41598-025-97362-z)
Supplement: Supplementary file 2 — Supplementary Material 2. [file 41598_2025_97362_MOESM2_ESM.pdf]

Long-read sequencing disentangles isoform complexity at allele-specific loci

Lison Lemoine[1,2], Sarah Hoelzl[1,2], Tim P. Hasenbein[1,2], Elisabeth Graf[3], Daniel Andergassen[1, 2*]

[1]Institute of Pharmacology and Toxicology, Technical University of Munich, Munich, Germany

[2]DZHK (German Centre for Cardiovascular Research), Partner Site Munich Heart Alliance, Munich, Germany

[3]Institute of Human Genetics, Klinikum rechts der Isar, Technical University of Munich, Munich, Germany

*daniel.andergassen@tum.edu

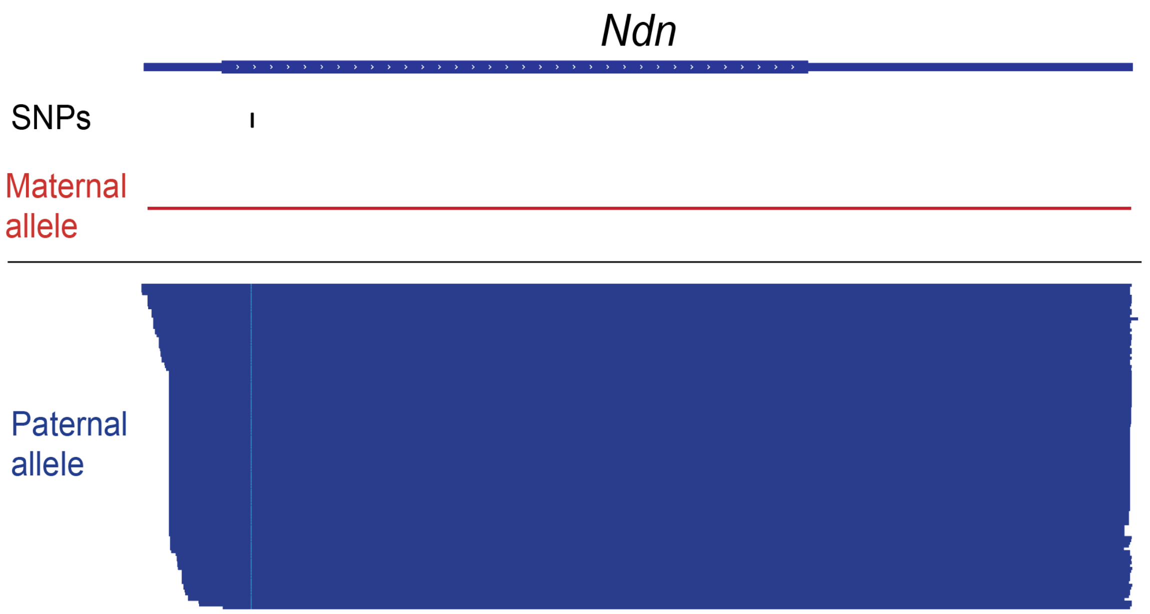

Supplementary Fig. 1. A single SNP per gene is sufficient to assign long reads to the corresponding allele. Shown is the paternally expressed imprinted *Ndn* gene correctly assigned by the WhatsHap tool using a single SNP.

Supplementary methods

Integrating PacBio Iso-Seq with whatshap phasing for allele-specific long-read analysis

1. Long-read Aligment

Raw reads were demultiplexed and processed using the standard PacBio pipeline (SMRT Link version 11.0). The align subcommand of Pbmm2 (version 1.10.0) is used to align the full-length non-concatemer reads (flnc.bam) to the reference genome (reference.fa). The output of this command is the file aligned.bam, which is sorted using the --sort flag.

```
$ pbmm2 align reference.fa flnc.bam aligned.bam --sort
```

Note: As described in the minimap2 documentation: By default, minimap2 assumes that the read orientation relative to the transcript strand is unknown. It tries two rounds of alignment to infer the orientation and write the strand to the ts tag if possible. Refer to https://github.com/PacificBiosciences/pbmm2 and https://github.com/lh3/minimap2 for more details.

2. WhatsHap phasing

The haplotag subcommand from whatshap (version 1.17) is used to tag the isoforms. The reference genome must be provided again, the reference index file must also be available in the reference folder and can be obtained using the faidx command from Samtools.

```
$ whatshap haplotag -o aligned_tagged.bam --reference reference.fa \
  snps.vcf.gz aligned.bam --ignore-linked-read --skip-missing-contigs \
  --output-haplotag-list haplotypes.tsv
```

SNP file

SNP information between multiple strains can be obtained from the Mouse Genomes Project (see Keane et al. Nature 2011). A VCF SNP file in the following tab-delimited format is required for Whatshap to map the reads to the corresponding allele. IMPORTANT: The file must be gzipped and include the index in the same folder that can be obtained using the tabix command from the HTSlib library.

```
##fileformat=VCFv4.1
#CHROM     POS   ID    REF   ALT   QUAL  FILTER    INFO  FORMAT    SAMPLE
chr1  3200405   .     T     A     50.0  .         .     GT        0|1
chr1  3200591   .     C     T     50.0  .         .     GT        0|1
chr1  3200708   .     A     C     50.0  .         .     GT        0|1
chr1  3200896   .     T     C     50.0  .         .     GT        0|1
chr1  3201035   .     A     C     50.0  .         .     GT        0|1
chr1  3201156   .     A     G     50.0  .         .     GT        0|1
chr1  3201267   .     G     A     50.0  .         .     GT        0|1
chr1  3201406   .     C     T     50.0  .         .     GT        0|1
```

There are two headers, the file format (here VCFv4.1) and the column names. Each column is mandatory. The first column is the chromosome name, followed by the position of the SNP. The columns ID, QUAL, FILTER and INFO are not necessary for the analysis and can be filled

with "." and a number for QUAL. The REF column represents the reference base (in this case Bl6) and the ALT column represents the non-reference allele (in this case CAST). The FORMAT column indicates what the format of the following column(s) will be. In this case, "GT" for the expected genotype, where 0 is the reference allele and 1 is the alternative allele. The pipe character (|) represents the phased genotype needed for this command.

The --ignore-linked-read flag disables the default feature that assigns the same haplotype to reads that belong to the same read cloud, and tags each one individually.

The --skip-missing-contigs flag ignores long reads that do not overlap with SNPs provided in the VCF file.

The most important output of this command is the haplotypes.tsv, which will be used in the next command. This file contains the read name, the assigned haplotype, the phase set, and the chromosome.

The split subcommand uses the haplotypes.tsv file to split the reads into two BAM files, one for each allele. Reads unable to be assigned to a haplotype will be discarded. The input BAM file here can be the original aligned BAM file or the haplotagged bam file.

```
$ whatshap split --output-h1 h1.bam --output-h2 h2.bam aligned.bam \
  haplotypes.tsv
```

3. Allelic long read counting

Finally, the number of reads per gene for each allele is counted using htseq-count from HTSeq (version 0.12.4), which allows us to calculate the allelic ratio. For this calculation, we divided the maternal reads by the total (maternal and paternal reads) for each gene.

```
$ htseq-count -s yes -r pos -f bam sorted_h1.bam annotation.gtf > h1.count
$ htseq-count -s yes -r pos -f bam sorted_h2.bam annotation.gtf > h2.count
```

This command requires an annotation file (GTF) and the BAM files to be sorted. These can be sorted using the Samtools sort command, which by default sorts by position.

The -r flag indicates how the files were sorted, in this case by position.

The -f flag specifies the file input, here BAM.

The -s flag indicates whether the data is stranded, and as explained above, long reads are stranded.
